# Prevalence and factors associated with anaemia in pregnant women in Cascades Region of Burkina Faso in 2012

**DOI:** 10.11604/pamj.2021.38.361.26612

**Published:** 2021-04-14

**Authors:** Bernard Ilboudo, Isidore Traoré, Clément Zemlé Méda, Alain Hien, Maurice Kinda, Michèle Dramaix-Wilmet, Gueswendé Blaise Léon Savadogo, Philippe Donnen

**Affiliations:** 1 Institut Supérieur des Sciences de la Santé, Université Nazi Boni, Bobo-Dioulasso, Burkina Faso,; 2 Institut National de Santé Publique, Ouagadougou, Burkina Faso,; 3 École de Santé Publique, Université Libre de Bruxelles, Bruxelles, Belgique

**Keywords:** Anaemia, pregnancy, Cascades region, Burkina Faso

## Abstract

**Introduction:**

anaemia in pregnancy is a public health concern in Burkina Faso. This study aims at identifying the factors associated with the prevalence of anaemia in pregnant women at a regional level in Burkina Faso.

**Methods:**

we conducted a cross-sectional study in the region called “Cascades”, located at the Western part of Burkina Faso. The study population included all pregnant women who attended antenatal care clinics in all public peripheral health facilities (districts) between May and June 2012 and agreed to participate in the study. They provided blood sample from which we measured the haemoglobin concentration using the Hemocue® system. The factors associated with anaemia in the study population were identified through multiple logistic regressions.

**Results:**

the prevalence (95% CI) of anaemia in pregnancy in the Cascades region was 58.9% (56.6% - 61.2%). Anaemia in pregnancy was more common in district of Banfora (OR = 1.40; 95% CI: 1.07-1.83), among housewives (OR = 2.96; 95% CI: 1.10-8.0), in the Mossi ethnic group (OR = 1.39; 95% CI: 1.04-1.85) and among the wives of farmers and artisans (OR = 2.55; 95% CI: 1.59-4.07). Anaemia in pregnancy was less frequent among women who drank local beer (OR = 0.68; 95% CI: 0.49-0.94).

**Conclusion:**

the prevalence of anaemia in pregnancy is high in Burkina Faso. Improving women's income level may contribute to reduce it. Further studies are needed to elucidate the link between the consumption of local beer, the local diet practices and anaemia in pregnant women.

## Introduction

Anaemia in pregnancy is a public health issue that affects both developed and low-income countries [1]. The World Health Organization (WHO) defines anaemia in pregnancy as a haemoglobin (hb) level lower than 11.0 g/dl at all gestational ages [2]. The main causes of anaemia in pregnancy frequently reported in studies conducted in Africa are nutritional deficiencies (especially iron and folic acid), haemorrhage during pregnancy, haemoglobinopathies, malaria, intestinal helminthiasis, HIV, *Helicobacter pylori* infections. Anaemia in pregnancy increases maternal and prenatal morbidity and mortality [3]. The factors most frequently associated with anaemia in pregnancy in Africa are multigestation, short interval between pregnancies, increased gestational age, rural residence, low education or income level, and insufficient preventive measures [4-8]. According to the results of the 2010 Demographic and Health Survey conducted in Burkina Faso, the prevalence of anaemia in pregnancy was 58% in Burkina Faso despite the implementation of recommended preventive strategies [9, 10]. Yet, no large-scale study has been published on the factors associated with this high prevalence of anaemia in pregnancy in Burkina Faso. The objective of this study was to determine the factors associated with the prevalence of anaemia in pregnant women at a regional level in Burkina Faso. It will provide opportunities for further research and help strengthen strategies to control anaemia in pregnant women.

## Methods

**The study type, location and period:** this was a facility-based cross-sectional study, carried out in the region called “Cascades”, located at the Western part of Burkina Faso. Data were gathered between May and June 2012. At that time, obstetric coverage exceeded 90% in the Cascades region, which had 77 public peripheral health facilities, one district hospital and one regional hospital.

**Study population and sampling:** all pregnant women who attended antenatal care clinics in all the public peripheral health facilities in the Cascades region during our study period and who agreed to participate in the study were included consecutively.

**Variables:** the dependent variable was anaemia in pregnancy, treated as a binary variable. It was defined by a haemoglobin level lower than 11 g/dl. The stages of anaemia were those of the WHO, categorized in 3 levels [1]: mild anaemia (hb: 10-10.9 g/dl), moderate anaemia (hb: 7-9.9 g/dl) and severe anaemia (hb < 7 g/dl). The independent variables were the respondent´s local health facility (district) (“Banfora”, “Mangodara” or “Sindou”), age in years categorised as adolescent girls (14-17 years), young adults (18-34 years) and adults (over 35 years), place of residence (rural or urban), woman´s occupation (housework, informal sector, salaried employment), spouse's occupation (agriculture, crafts, animal farming, trade, salaried employment) woman´s marital status (married or single woman), education level (unschooled, primary, secondary or higher), ethnic group (native, Mossi, Fulani, Lobi or other), religion (Islam, Christianity, traditional religion or other), distance to health facility in kilometres (≤ 5, 6-10, > 10), availability of a mosquito net, consumption of local alcohol, coffee or tea, geophagy, number of antenatal visits, age (in years) at first pregnancy categorised according to its quartiles, gravidity grouped into low gravidity (1 to 2 pregnancies) and multigravidas (more than 2 pregnancies), the gestational age in trimesters, the age of the last child in years to reflect the last pregnancy interval and categorised as reduced (0 to 2 years), ideal (3 years) and large (over 3 years), weight in kilograms categorised by the median, iron/folic acid supplementation and preventive treatment of malaria (in number of doses of *sulfadoxin-pyrimethamine*).

**Data collection:** data were gathered from pregnant women using a standardised questionnaire. The woman's age, weight and gestational age were reported from their health booklets. Due to the low educational level of the participants, the interview was conducted orally in the local language (*Dioula*). The haemoglobin level was measured with devices using the Hemocue® - Hb 301 system technique [11]. The questionnaire was validated by specialists in public health, epidemiology and biostatistics from the *Université libre de Bruxelles* in Belgium and the *Université Nazi Boni* in Burkina Faso. The data was collected by trained health workers under the supervision of the research team. The mean altitude of the Cascades region in relation to the sea level did not require adjustment of haemoglobin levels [2].

**Bias management:** to reduce the bias due to the language barrier, the questionnaire was translated into *Dioula*, the main local language in the region. A pre-test of the questionnaire was carried out in the neighbouring region called “Hauts-Bassins” before the survey began. The questions were clarified with the data collectors in order to harmonise their understanding. A meeting was held with them at the end of the first day of data collection, then once a week to assess and correct the completion of the tools. At the beginning of the survey, and then once a week, all haemoglobinometers were checked using an automated haematology device. The data collectors and the pregnant women included in the study received a code to avoid misclassification. The gestational age was estimated by the prenatal health care providers from the dates of the last menstrual period and compared to the measurement of fundal height. All data were visually controlled daily by the field supervisor. Data entry was duplicated to reduce errors.

**Data processing and analysis:** the data were analysed using SPSS IBM-Statistics® 24 and STATA-SE® 16. All variables were summarised using descriptive statistics (numbers and percentages). The association between the independent variables and anaemia was assessed by univariate analysis using the chi-square test. The Odds Ratios (OR) followed by the 95% confidence interval (95% CI) and p-value were determined using logistic regression. The variables with a p-value of less than 20% were considered in a multiple logistic regression model and selected for the final model by a stepwise digressive procedure. We also performed stratification analysis to identify factors associated with anaemia by district. The adjustment of the logistic regression models was verified by the Hosmer and Lemeshow test. Only variables that were statistically significant at the 5% threshold were retained in the final models from which adjusted Odds Ratios and their 95% confidence intervals were reported.

**Ethics:** the study was conducted in accordance with the terms of the Declaration of Helsinki [12] and all procedures involving the participants were approved by the Ethics Committee of the *Centre Muraz* (Ref. 022-2012/CE-CM, 22-02-2012) in Bobo-Dioulasso, Burkina Faso. The study has also been approved by the health authorities. Informed and written consent was obtained from all participants. Pregnant women suffering from anaemia (haemoglobin level < 11 g/dl) were managed as part of the antenatal program and received iron and folate supplementation. The study did not involve any major risks to the integrity and rights of the participants.

## Results

**Study population and prevalence of anaemia:** a total of 1763 pregnant women were included in the study in all 77 health facilities of the Cascades region. Among them, the prevalence of anaemia was 58.9% (95% CI: 56.6% - 61.2%). Anaemia was mild in 31.1% of women, moderate in 26.0% and severe in 1.9%. Table 1 presents a description of pregnant women characteristics and their association with anaemia. The district of Banfora accounted for almost half of the women included. The average age of the women was 26.1 (6.3) years ranging from 14 to 48 years. The average age at first pregnancy was 18.1 (2.8) years ranging from 11 to 31 years. The majority of women lived in rural areas, were housewives, married or in common law unions, unschooled, Muslim, in their 2^nd^ or 3^rd^ trimester of pregnancy. Their spouses were mostly farmers or artisans. The dominant ethnic group was composed of native ethnic groups (Goin, Karaboro, Senoufo, Turka and related). Two-thirds of the women lived within 5 kilometres of a health facility.

**Table 1:** the socio-demographic characteristics of pregnant women in 2012 in the Cascades region of Burkina Faso and their association with anaemia in pregnancy

Variable	n	%	% anaemia	OR (CI95%)	p
**District**					0.081
Banfora	848	48.1	60.3	1.30 (1.02-1.66)	
Mangodara	539	30.6	60.3	1.30 (1.00-1.70)	
Sindou	376	21.3	53.9	1	
**Woman´s occupation**					<0.001
Housework	1585	92.6	60.6	5.34 (2.16-13.40)	
Informal sector	99	5.8	50.0	3.50 (1.30-9.42)	
Employee	27	1.6	22.2	1	
**Spouse´s occupation**					<0.001
Cotton grower/craftsman	382	22.2	63.8	2.57 (1.71-3.88)	
Other farmer	1001	58.2	58.7	2.08 (1.42-3.02)	
Trader	136	7.9	58.5	2.06 (1.26-3.37)	
Animal farmer	73	4.3	71.2	3.62 (1.95-6.71)	
Employee	128	7.4	40.6	1	
**Education**					<0.001
Unschooled	1408	80.3	61.2	2.02 (1.37-2.97)	
Alphabetised	40	2.3	45.0	1.05 (0.51-2.16)	
Primary	192	10.9	55.3	1.58 (0.99-2.52)	
Secondary or High school	114	6.5	43.9	1	
**Ethnic group**					0.003
Native	1000	56.9	57.4	1	
Fulani	133	7.7	67.7	1.56 (1.06-2.29)	
Mossi	357	20.3	65.3	1.40 (1.09-1.80)	
Lobi	132	7.5	52.3	0.81 (0.57-1.17)	
Other	134	7.6	52.3	0.81 (0.57-1.17)	
**Religion**					0.019
Muslim	1382	81.6	60.5	1	
Catholic	139	8.2	48.2	0.61 (0.43-0.86)	
Protestant	32	1.9	62.5	1.10 (0.53-2.24)	
Traditional	120	7.1	50.4	0.66 (0.46-0.97)	
Other	21	1.2	57.1	0.81 (0.36-2.08)	
**Local alcohol consumption**					0.007
Yes	189	10.7	49.7	0.66 (0.49-0.89)	
No	1574	89.3	60.0	1	
**Age at 1^st^ pregnancy (years)**					0.008
11-16	453	26.7	58.7	1.33 (1.01-1.75)	
17	334	19.7	63.2	1.60 (1.18-2.16)	
18-19	538	31.7	61.3	1.48 (1.13-1.93)	
20-42	375	22.1	51.7	1	
**Gestational age**					0.052
First quarter	153	8.8	50.0	1	
Second quarter	670	38.6	59.0	1.44 (1.01-2.05)	
Third quarter	913	52.6	60.5	1.53 (1.08-2.16)	
**Weight (kg)**					0.006
<53	348	20.1	65.5	1.41 (1.10-1.80)	
≥53	1384	79.9	57.4	1	
**Gravidity**					0.063
1-2	617	35.6	55.9	1	
3+	1114	64.4	60.5	1.21 (0.99-1.47)	
**Coffee consumption**					0.107
Every day	256	14.5	64.7	1.29 (0.96-1.73)	
Sometimes	790	44.8	57.2	0.94 (0.77-1.16)	
Never	717	40.7	58.7	1	

In univariate analysis, the frequency of anaemia in pregnancy was significantly higher among housewives and women working in the informal sector, unschooled women, Fulani and Mossi ethnic groups, those weighing less than 53 kg and those who had their first pregnancy before the age of 20. The prevalence of anaemia was significantly lower among salaried women, wives of salaried employees, women who believed in traditional religion and those who consumed alcohol (local beer).

Anaemia in pregnancy appeared to be more frequent in the districts of “Banfora” and “Mangodara”, in the 2^nd^ and 3^rd^ trimesters of pregnancy, among women living beyond 10 km from a health facility or who had received less than 2 doses of *sulfadoxin-pyrimethamine*, but the association was not significant. Age, residence, marital status, the use of mosquito “bed-net”, coffee/tea consumption, geophagy, number of antenatal visits, gestational age, age of last child and iron/folic acid supplementation were not associated with anaemia in pregnancy in univariate analysis.

**Factors associated with anaemia in pregnancy in multivariate analysis**: Table 2 presents the factors independently associated with anaemia in pregnancy in the multiple logistic regression model for the whole sample. The prevalence of anaemia was significantly higher in the district of “Banfora”, among housewives, Mossi ethnic group and wives of farmers, artisans and traders. It was significantly lower among women who drank local alcohol. In an analysis of the interactions between women's characteristics and the district, alcohol consumption was associated with a low frequency of anaemia only in “Mangodara” district; coffee and tea consumption, as well as low age at first pregnancy and gravidity greater than 2 were associated with a higher frequency of anaemia, but only in “Banfora” district. There was no statistically significant interaction between the other characteristics of pregnant women and the district on the prevalence of anaemia (Table 3).

**Table 2:** multivariate analysis of factors associated with anaemia in pregnancy in the Cascades region (n = 1669 including 990 cases of anaemia)

Variable	Adjusted OR (CI95%) *	p
**District**		0.034
Banfora	1.40 (1.07-1.83)	
Mangodara	1.16 (0.84-1.60)	
Sindou	1	
**Occupation of women**		0.042
Housework	2.96 (1.10-8.00)	
Informal sector	2.06 (0.72-5.84)	
Employee	1	
**Spouse´s occupation**		0.002
Cotton grower/craftsman	2.55 (1.59-4.07)	
Other farmer	1.82 (1.17-2.81)	
Trader	1.78 (1.05-3.01)	
Animal farmer	2.25 (0.96-5.28)	
Employee	1	
**Ethnic group**		0.039
Native	1	
Fulani	1.41 (0.79-2.52)	
Mossi	1.39 (1.04-1.85)	
Lobi	0.84 (0.56-1.26)	
Other	0.84 (0.57-1.24)	
**Local alcohol consumption**		0.021
Yes No	0.68 (0.49-0.94)	


* Hosmer-Lemeshow test: p = 0.478; * adjustment variables: education, religion, distance to health centre, net ownership, age at 1st pregnancy, gestational age, weight, sulfadoxin-pyrimethamine intake

**Table 3:** association between anaemia and the characteristics of pregnant women by district

Variables	Banfora OR (CI95%)	Mangodara OR (CI95%)	Sindou OR (CI95%)	P interaction
**Age (years)**				0.883
14-17	1.31 (0.67-2.54)	1.06 (0.51-2.23)	0.76 (0.35-1.65)	
18-34	1	1	1	
35 +	1.17 (0.75-1.81	1.06 (0.55-2.07)	1.17 (0.63-2.15)	
**Occupation of women**				0.822
Housework	7.13 (2.37-21.40)		5.09 (0.56-46.00)	
Informal sector	5.21 (1.59-17.09)	NA	2.67 (0.24-30.07)	
Employee	1		1	
**Spouse´s occupation**				0.161
Cotton grower/craftsman	4.42 (2.58-7.55)	0.67 (0.22-1.96)	2.13 (0.78-5.85)	
Other farmer	2.64 (1.66-4.20)	0.74 (0.27-2.02)	1.86 (0.69-5.01)	
Trader	2.42 (1.31-4.47)	0.89 (0.27-2.90)	1.57 (0.40-6.14)	
Animal farmer	6.26 (2.30-17.05)	1.14 (0.34-3.81)	2.36 (0.49-11.45)	
Employee	1	1	1	
**Ethnic group**				0.438
Native	1	1	1	
Fulani	1.66 (0.90-3.05)	1.70 (0.90-3.23)	0.99 (0.38-2.58)	
Mossi	1.68 (1.13-2.50)	1.30 (0.83-2.02)	0.16 (0.02-1.37)	
Lobi	0.83 (0.39-1.77)	0.85 (0.51-1.43)	0.26 (0.03-2.57)	
Other	0.77 (0.44-1.35)	1.16 (0.53-2.52)	0.69 (0.37-1.30)	
**Local alcohol consumption**				0.019
Yes	0.71 (0.49-1.05)	0.18 (0.07-0.50)	0.98 (0.52-1.86)	
No	1	1	1	
**Coffee consumption**				0.003
Every day	2.64 (1.62-4.29)	0.77 (0.43-1.36)	0.64 (031-1.32)	
Sometimes	1.25 (0.93-1.68)	0.99 (0.65-1.50)	0.50 (0.27-0.95)	
Never	1	1	1	
**Tea consumption**				0.010
Every day	2.32 (0.97-5.56)	0.70 (0.32-1.56)	1.42 (0.54-3.73)	
Sometimes	1.37 (1.03-1.82)	0.67 (0.47-0.97)	0.76 (0.50-1.16)	
Never	1	1	1	
**Age at 1^st^ pregnancy (years)**				0.053
11-16	2.03 (1.34-3.10)	0.90 (0.54-1.50)	0.92 (0.51-1.65)	
17	2.57 (1.67-3.96)	0.96 (0.54-1.73)	1.03 (0.55-1.93)	
18-19	1.83 (1.27-2.63)	1.03 (0.61-1.75)	1.29 (0.72-2.33)	
20-42	1	1	1	
**Gravidity**				0.030
1-2	1	1	1	
3+	1.51 (1.13-2.02)	0.81 (0.56-1.17)	1.30 (0.84-2.0)	
**Gestational age**				0.720
First quarter	1	1	1	
Second quarter	1.59 (0.97-2.61)	1.32 (0.68-2.54)	1.39 (0.62-3.12)	
Third quarter	1.68 (1.04-2.72)	1.20 (0.63-2.25)	1.81 (0.81-4.04)	
**Iron supplementation**				0.620
Yes	1	1	1	
No	1.01 (0.71-1.43)	1.40 (0.80-2.46)	1.05 (0.58-1.90)	
No of doses of SP*				0.354
0	1.47 (0.29-7.39)	8.94 (1.03-77.8)		
1	1.44 (0.28-7.43)	9.53 (1.07-85.10)	NA	
2	1.40 (0.27-7.09)	5.80 (0.66-50.69)		
3 +	1	1		

NA: Not applicable due to low numbers in some categories; *Sulfadoxin-pyrimethamine

## Discussion

Our results confirmed the high prevalence of anaemia in pregnancy in Burkina Faso; it exceeded the severity threshold of 40% established by the WHO [1]. This is the first large-scale study devoted to identify factors associated with anaemia in pregnancy in Burkina Faso. It covered a wide range of factors and all peripheral antenatal clinics of the Cascades region with an enlarged sample to compensate for the influence of missing data on the accuracy of the results. The analysis of health statistics and previous studies carried out in Burkina Faso, compared to our results, reveals that the prevalence of anaemia in pregnancy in our country was stable. The WHO estimated it at 68.3% between 1993 and 2005, then 58.0% in 2011 [1, 13]. Méda *et al*. estimated it at 66.0% in 1996 and 63.1% in 2010 in studies conducted at district-level on patients with HIV infection [5, 14]. The regional prevalence found in our study was closer to the results of the 2010 National Demographic and Health Survey reported by WHO in 2011 [9]. This similarity confirms a fairly good representativeness of our sample. In 2015, in a community trial which tested the effect of a personalised support for pregnant women, the baseline prevalence of anaemia in pregnancy was 63.3% (Ilboudo B, Savadogo LGB, Traoré I *et al*. Effect of a personalized support for pregnant women on the prevalence of anaemia in pregnancy in Burkina Faso, accepted under No. AJTMH 20-1043-R1 in process of publication). The prevalence of anaemia in pregnancy has therefore not declined for three decades in Burkina Faso. This justifies the need to improve strategies for the prevention of anaemia in pregnancy, based among other things on the use of research findings.

According to estimates published by the WHO in 2016, the prevalence of anaemia in pregnancy in West African countries was close to the prevalence we reported in our study; it ranged from 42.9% (20.9-63.3) in Cape Verde to 61.4% (51.7-67.1) in Togo, where the prevalence was the highest in the African continent [1]. But the prevalence published by the authors of these countries are sometimes distant from WHO estimates. Nearly all of the studies involved women attending antenatal clinics and the prevalence varied over the same time period within the same country depending on the study site. With five-year period 2015-2019 data from Ghana, Anlaakuu *et al*. found a prevalence of anaemia of 40.8% in the Brong Ahafo Region, compared to a prevalence of 56% found by Tibambuya *et al*. in the Upper West Region. In these studies, women were enrolled in only one health facility [6, 15]. In a study conducted by Sholeye *et al*. in 4 peripheral health centres of Ogun State in south-western Nigeria in 2013, the prevalence of anaemia in pregnancy was 32%, much lower than what we found. This study was conducted in an urban setting and included only mono-foetal pregnancies, without women with any morbidity including haematological disorders [16]. In the multi-country trial conducted between 2013 and 2015 by the COSMIC Consortium in Burkina Faso, Benin and The Gambia, the pre-intervention prevalence of anaemia in pregnancy was 43% for all three countries, well below the sub-regional average prevalence of 56% estimated by the WHO [17, 18]. No reviewed [17, 18]. No review articles reconciling these findings have been published on the prevalence of anaemia in pregnancy and the associated factors in West Africa.

Globally, according to the 2016 WHO estimates, the prevalence of anaemia in pregnancy ranged from 16.2% in the USA to 63.0% in Yemen. The African continent and South Asia were the regions with the highest prevalence of anaemia in pregnancy [18]. Some systematic review articles and meta-analyses have found the same tendencies of prevalence [19, 20].

In our study, five factors were independently associated with the high prevalence of anaemia in pregnancy at the regional level: living in the district of Banfora, the low-income level of the pregnant woman or her spouse, being from the Mossi ethnic group and not consuming the local alcohol. The analysis of interactions with the district showed that alcohol consumption was associated with a low frequency of anaemia only in Mangodara district; coffee and tea consumption, young age at first pregnancy and gravidity were associated with the high frequency of anaemia, but only in the district of Banfora. The prevalence of anaemia was higher in the district of Banfora district than in the other two districts. This could be explained by the fact that the district of Banfora multiple factors showed association with a higher frequency of anaemia in pregnancy (coffee, tea, young age at first pregnancy and multigestation); on the other hand, this district had the lowest indicators of obstetric care coverage in the region [21].

Compared to employed women, housewives had a higher prevalence of anaemia. About this, some authors have shown that the level of personal income of pregnant women influences their nutritional status [22]. The women with their own income are able to afford better care, to choose and diversify their diet, especially meat, fish, eggs and offal, which are some foods very rich in bioavailable iron, which is useful in preventing anaemia in pregnancy. Anaemia in pregnancy was also less common among the wives of salaried employees. Since employment is correlated with education and income level, wage employment seemed to be a guarantee of autonomy that promotes better monitoring of pregnancy and a balanced diet for pregnant women, especially since the majority of the population was unschooled and the main occupation was non-mechanized subsistence farming. Among ethnic groups, the Mossi had the highest frequency of anaemia in pregnancy compared to the native group. Although this was not statistically significant, the Fulani ethnic group had higher anaemia frequency. This difference in the frequency of anaemia in pregnancy between ethnic groups was probably related to lifestyle and eating habits specific to ethnic groups, which also deserve further investigations.

The consumption of local alcohol was associated with a lower frequency of anaemia in pregnancy. In subgroup analysis this association was statistically significant in the district of Mangodara but not in the other two districts. There were no plausible factors explaining this result, which requires further investigations to understand the mechanism. Determining the composition and quantities of the different alcoholic beverages consumed by women from this district, as well as the possible confounding factors could contribute to explain these results related to alcohol. Intermittent preventive treatment (IPT) of malaria with *sulfadoxin-pyrimethamine* was not associated with anaemia in pregnancy. Malaria is one of the major causes of anaemia in endemic countries such as Burkina Faso where its incidence reached 39 per 1000 pregnant women per month in 2012 [23]. IPT was recommended by the WHO and introduced in Burkina Faso in 2006 after evidence of the failure of chloroquine to prevent malaria in pregnant women. It was possible that confounding factors may have masked the association between IPT, the use of mosquito bed-net and iron supplementation following anaemia in pregnancy, as many authors have logically observed [24, 25].

The only published studies on factors associated with the prevalence of anaemia among women attending antenatal clinics in Burkina Faso were conducted by Meda *et al*. in 1995-96 in the urban area of Bobo-Dioulasso and in 2010 in the health district of Houndé. In the 1996 study, they concluded that anaemia in pregnancy was associated with HIV infection, advanced gestational age and women with low socio-economic status. The study was carried out in an urban setting and in a context of prevention of mother-to-child transmission of human immunodeficiency virus (HIV), with an HIV prevalence of over 9% among pregnant women [14]. In the 2010 study conducted in a rural district, in addition to advanced gestational age, geophagy, low educational attainment and multigestation were associated with a higher frequency of anaemia in pregnancy. In this study, infections were not associated with anaemia.

Our results confirmed the association between anaemia in pregnancy and the factors identified by Meda *et al*. with exception for HIV infection and geophagy. This could be explained by the low relative prevalence of HIV infection, which was below 1% among pregnant women in 2012 in the Cascades region [26]. Also, there was no association between anaemia and a history of fever (which is a sign of HIV infection) in our study. Elsewhere in the world, the factors most often associated with a higher frequency of anaemia in pregnancy are malnutrition (low mid-upper arm circumference, weight loss or iron deficiency) [6, 15, 16, 27, 28], low socio-economic or schooling level [16, 27-31], malaria [6-8], multigestation [7, 27] and health facility [30, 31]. Some of these factors were associated with anaemia in our study (income level, district).

According to data published in the literature, alcohol consumption reduces the risk of iron deficiency anaemia, but exposes the consumer to the consequences of hemosiderosis [32]. The mechanism stems from the increased activity of aspartate aminotransferase linked to alcohol-induced liver damage. This may explain the reduction in the frequency of anaemia in pregnancy among local alcohol consumers in our study, particularly in the district of Mangodara where, more than elsewhere, addiction to local beer was a practice rooted in local culture [33]. Nevertheless, there are confounding factors that have not been elucidated which could help explain the relation between local alcohol consumption and anaemia. It is unanimously recognised in the literature that advanced gestational age is associated with a greater frequency of anaemia, but it is most often described as a confounding factor depending on the covariates considered in the study [5, 14, 27, 31, 34]. In our case, the gestational age was associated with anaemia in univariate analysis but did not contribute to the final model.

The literature has also established the link between the increased frequency of iron deficiency anaemia and the consumption of coffee and tea [29, 35]. This supports the results of our subgroup analysis where coffee and tea consumption were associated with a high frequency of anaemia in pregnancy, particularly in the district of Banfora. However, the association varied greatly from one district to another and was sometimes even reversed, raising suspicion of the influence of confounding factors that have not been elucidated. Analysis that includes age and marital status did not show an association between these two factors and anaemia in pregnancy [5, 8, 30]. All these observations confirm that anaemia in pregnancy remains a public health issue in Burkina Faso and many contextual factors determine its prevalence.

The main limitation of the study was inherent to the selection of pregnant women attending antenatal clinics and not in the community. Nevertheless, given the high attendance of prenatal care within pregnant women [21], our study sample was sufficiently representative of the population of pregnant women in the region. Further studies, taking into account the factors not elucidated in our study, will be useful to deepen the explanation of the high prevalence of anaemia in pregnancy.

## Conclusion

The prevalence of anaemia in pregnancy was high in the Cascades region of Burkina Faso. The main factors associated with this high prevalence were reported from unemployed woman as well as those with unemployed spouse, women from the Mossi ethnic group, those not consuming local alcohol and those living in the district of Banfora. Further studies will be useful to clarify the relationship between anaemia in pregnancy and local eating habits. Improved strategies to reduce the prevalence of anaemia in pregnancy taking into account the identified factors would be beneficial in Burkina Faso.

### What is known about this topic

- Anaemia in pregnancy is a public health concern in Burkina Faso;
- Burkina Faso has implemented the recommended strategies to reduce the prevalence of anaemia in pregnancy.

### What this study adds

- The prevalence of anaemia in pregnancy has not declined in Burkina Faso for three decades despite the application of the recommended guidelines;
- Factors associated with high prevalence of anaemia in pregnancy are mainly socio-cultural;
- Women who drink the local beer are less likely to show anaemia in pregnancy.
